# Research Progress on the Mechanism of Lumbarmultifidus Injury and Degeneration

**DOI:** 10.1155/2021/6629037

**Published:** 2021-02-26

**Authors:** Xianzheng Wang, Rui Jia, Jiaqi Li, Yibo Zhu, Huanan Liu, Weijian Wang, Yapeng Sun, Fei Zhang, Lei Guo, Wei Zhang

**Affiliations:** ^1^Department of Spinal Surgery, The Third Hospital of Hebei Medical University, Shijiazhuang, 050000 Hebei Province, China; ^2^Department of Reproductive Medicine, The Second Hospital of Hebei Medical University, China; ^3^School of Chemical Engineering, The University of Queensland, Australia

## Abstract

This review summarizes recent research progress in the clinical features, image manifestations, and pathological mechanism of multifidus injury. After a brief introduction to the fiber classification, innervation, blood supply, and multifidus function, some factors of multifidus injury, consisting of denervation, intraoperative incision selection and traction, and lumbar degenerative disease are overviewed. In addition, the clinical index of multifidus injury including myoglobin, creatine kinase, IL-6, C-reactive protein, the cross-sectional area of multifidus, the degree of fat infiltration, and intraoperative biopsy are summarized. Furthermore, we recommend that patients with chronic low back pain should take the long-term exercise of lumbodorsal muscles. Finally, some remaining issues, including external fixation and the imaging quantitative evaluation criteria of multifidus, need to be further explored in the future.

## 1. Introduction

All around the globe, 65-85% of the population suffer from low back pain, which is highly related to lumbar degenerative disease [1, 2]. The recurrent low back pain not only brings the loss of productivity and working time but also increases the economic burden borne by the whole society [3]. Studies showed that most patients with lumbar degenerative disease had atrophy of multifidus muscle and fat infiltration [4, 5].

Multifidus is the only paraspinal lumbar muscle innervated by a single nerve root [6], which plays an important role in maintaining the stability of the spine [7]. Multifidus injury often occurs in patients with chronic low back pain, lumbar disc herniation, and scoliosis and lumbar surgery [8–10]. Multifidus injury is often manifested as atrophy and steatosis in imaging and increases inflammatory reaction [11]. In the development of lumbar degenerative diseases, the protection and treatment of multifidus need more interventions.

In this study, we try to review the anatomical structure and function of multifidus, the clinical manifestations, imaging findings, pathological mechanism, and research progress of multifidus injury; discuss the risk factors; list the clinical detection methods of multifidus injury; explore the mechanism of oxidative stress that induced multifidus injury; and finally, we summarized the recovery methods of multifidus injury, which may play a positive role in clinical work.

## 2. Anatomy and Function of Multifidus

Multifidus is the deep internal back muscle. It is composed of multiple muscle bundles and fills the groove on both sides of the spinous process. It is close to the innermost side of the spine and has the largest attachment area in the paravertebral muscle. The lumbar multifidus is wrapped in the muscle sheath formed by the superficial and middle layers of the thoracolumbar fascia. The Longissimus muscle, spinous process, and lamina are on the outside, medial side, and the ventral side of the multifidus.

The multifidus muscle originates from the sacrum and the posterior superior iliac spine, the mastoid process of the lumbar spine, the transverse process of the thoracic vertebrae, and the articular process of C4-C7. It attaches to all the spinous processes of the upper vertebrae. Rosatelli et al. [12] found that L1-L4 multifidus could be divided into three layers: shallow and middle and deep layer. L5 multifidus was divided into two layers, shallow layer and deep layer. The superficial multifidus started from the L1-L5 spinous process and moved outward and downward. L1 multifidus stopped at L5, S1 mastoid, and posterior superior iliac spine; L2 multifidus stops at S1 mastoid and posterior superior iliac spine; L3 multifidus stops at S1-S3 dorsal side; L4 multifidus stops at the dorsal side of S2-S4; L5 multifidus stops at the dorsal side of S3-S4. The medial multifidus starts from the L1-L4 spinous process and ends at the L4, L5, S1 mastoid, and dorsal side of S2. The deep multifidus starts from the L1-L5 lamina and ends at the L3-S1 mastoid and sacrum.

Multifidus muscle fibers are classified as type I fibers (slow-twitch fibers), type IIa fiber (fast-twitch fiber), and type IIX fiber (fast-twitch glycolytic) [13, 14]. Type I fiber has slow contraction speed and small contraction strength, but its antifatigue ability makes it work for a long time. It possesses low ATPase activity, low maximal velocity, higher mitochondrial content, and more significant oxidative enzyme complement than the type II fiber. The aerobic capacity of type I fiber is higher than that of type II fiber. Type II fiber has short contraction latency, fast contraction speed, strong contraction, and weak force fatigue resistance. Type II fibers are endowed with higher ATPase activities and contain enzymes, which support the regeneration of ATP through anaerobic mechanisms. The size of type II muscle fiber decreased significantly with the increase of adult age, while type I muscle fiber was the opposite [15]. In the elderly, type II muscle fiber size is significantly smaller than that of young people [16]. Some studies have shown that the lumbar dorsal multifidus muscles consist of 54%–70% type I fibers and 23.6%–52.54% type II fibers [17]. The atrophy of type I fibers in the multifidus muscle can be explained by muscle fibers chronic stretch due to pain-induced spasm. Moreover, the atrophy of type II fibers was often attributed to low activity level [18].

The lumbosacral multifidus is innervated by the dorsal root of the lumbar nerve [19], which divides from the spinal nerve [20, 21] and passes through a bone fiber ring to the medial edge of the intertransverse muscles, and is divided into the medial and lateral branch. The medial branch runs backward and downward at the lateral side of the lower vertebrae, passes through the bone fiber tube, reaches the dorsal side of the lamina, and then enters the multifidus muscle. However, due to the small anatomic structure and lack of elasticity, the stenosis of for a man can oppress the lumbar nerve and cause low back pain.

The lumbosacral multifidus is supplied by the dorsal branch of the lumbar artery [22] and the lateral iliac artery. From both sides of the abdominal aorta, the lumbar artery crosses the anterior and lateral sides of the lumbar vertebral body and accompanies the lumbar vein. It divides the dorsal branch at the medial edge of the psoas major to supply the multifidus and other dorsal muscles [23].

**Figure 1:**
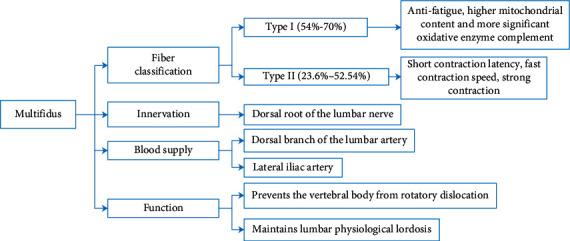
Anatomy and function of multifidus.

In paravertebral muscles, multifidus is the primary source of maintaining lumbar stability [24]. The superficial multifidus muscle crosses multiple segments, moves outward and downward from the L1-L5 spinous process, prevents the vertebral body from rotatory dislocation, and maintains lumbar physiological lordosis. The deep multifidus muscle crosses few segments and is close to the central axis, which can increase lumbar segmental tension and reduces the movement between lumbar segments [25]. Some scholars believe that when the body's centre of gravity suddenly loses balance, the multifidus will be activated in advance and contract ahead of time enhancing the stability of the lumbar spine sequence, which is called feedforward control [26, 27]. Due to the short length, large cross-sectional area, and the short reaction time of multifidus, the risk of lumbar instability can be reduced (Figure 1).

## 3. Factors of Multifidus Injury

### 3.1. Denervation

Lumbar degenerative diseases are often related to nerve injury, and most of the electromyography shows potential denervated changes. In the experiment, the multifidus muscle of pig atrophied rapidly after nerve root injury [28]. There are a lot of pathological changes in denervated multifidus: reduced diameter of muscle fiber [29] and cross-sectional area of muscle bundle [30], dissolved muscle fiber mitochondria [31], and disordered transverse tubular system. As the number of mitochondria decreased, the sodium-potassium pump activity decreased, the energy supply of the tricarboxylic acid cycle decreased, and muscle strength weakened. Also, the absence of proprioceptor in multifidus will lead to the interruption of feedforward reflex and a further decrease of spinal stability. Some scholars have pointed out [32] that asymmetric multifidus atrophy will break the biomechanical balance of the spine and cause spinal degeneration. Fortunately, Cha et al. [33] pointed out that patients with preoperative denervation of the multifidus had reinnervation of the multifidus during a 12-month follow-up after bone graft fusion, which meant that nerve recovery after the operation was possible.

### 3.2. Intraoperative Incision Selection and Traction

During the posterior lumbar surgery exposure process, it is inevitable that paravertebral muscles will be stripped and damaged [34]. Postoperative lumbar MRI shows that paravertebral muscles have different degrees of injury [35], accompanied by low back pain and dysfunction [36]. However, minimally invasive surgical methods can significantly reduce paravertebral muscle atrophy. Studies have shown that the paramedian approach is used to separate the multifidus and the longissimus muscle in the posterior lumbar approach. The postoperative multifidus atrophy is 4.8%, significantly less than the posterior median approach (20.7%) [37].

Posterior lumbar interbody fusion is an important cause of multifidus muscle injury and atrophy in posterior lumbar surgery [38]. Fusion and fixation of the posterior lumbar spine usually requires extensive anatomy and forced traction of the paravertebral muscles, which may seriously damage the structure and function of the muscles. Denervation and abandonment may be important factors of multifidus atrophy [38]. The underlying pathophysiology of muscle injury may involve mechanical and ischemic mechanisms. On the other hand, surgical related stress may trigger some protective responses in the injured paraspinal muscles. After the contraction of multifidus, the expression of heat shock protein 70 and malondialdehyde was significantly increased. Through the study of multifidus samples, Lu et al. found that the decrease of heat shock protein 70 in muscle cells after the long-term contraction resulted from severe muscle injury [39].

Kawaguchi et al. [40] pointed out that the traction of paravertebral muscles or excessive pressure on the lumbodorsal muscles during the operation will induce the injury and bleeding, and the length of the traction time also affects the degree of atrophy. Some researchers [41] suggest that the operator should perform a 5-minute stretch release every hour to avoid severe muscle injury after surgery. However, direct intraoperative injury is not the only factor for multifidus atrophy. Motosuneya et al. [42] found that although the lumbodorsal muscles of patients in the anterior lumbar interbody fusion group did not undergo surgical trauma, paravertebral muscle atrophy still occurred after surgery. It is pointed out that the use of external fixation such as waist supporter after operation leads to decreased activity of the operative segment, which is the main cause of paravertebral muscle injury in patients with anterior lumbar interbody fusion.

### 3.3. Lumbar Degenerative Disease

Lumbar degenerative disease is one of the causes of multifidus injury [43]. After multifidus injury, the biomechanical balance of the spine is disturbed. The frequent displacement between segments will accelerate the degeneration of the spine, which will lead to further injury of paraspinal muscles. Furthermore, if no effective intervention is taken, a vicious circle will be formed. Faur et al.'s study showed a significant correlation between lumbar disc degeneration and fat atrophy of multifidus, and at the level of L5/S1, the percentage of multifidus atrophy was higher than other segments [30]. The causes of lumbar degenerative disease leading to multifidus atrophy can be divided into two aspects: (1) compression caused by lateral recess stenosis [44], intervertebral disc herniation, long-term local ischemia, and nerve damage leading to denervated atrophy [45]. (2) The sinuvertebral nerve compressed unilaterally causes low back pain [46], and the reduced exercise of the affected side leads to disuse atrophy of multifidus muscle. Wan et al. [47] found that the multifidus on the affected side of patients with chronic low back pain was significantly atrophied compared with the healthy side. On the contrary, Ranger et al. [48] believed a negative correlation between multifidus atrophy and chronic low back pain after 12 months of follow-up, which means the relationship between paravertebral muscles and chronic low back pain still needs to be confirmed by a higher quality cohort study.

Chronic low back pain is one of the most common and costly medical problems; very few treatments have proved effective [49, 50]. However, very few treatments have proved effective. Among the 291 diseases studied, lower back pain in Western Europe was ranked as the highest disability burden, according to the Agten et al.'s study [51]. Rahmani et al.'s study showed that pain intensity and disability index were significantly correlated with muscle size, and the multifidus muscle size of 15-18 years old male adolescents with low back pain was lower than that of healthy people [37].

## 4. Oxidative Stress and Inflammation of Multifidus

**Figure 2:**
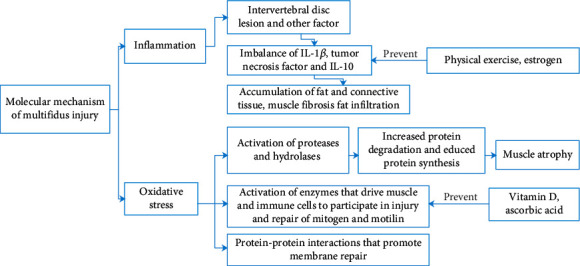
Molecular mechanism of multifidus injury. IL: interleukin.

Oxidative stress and inflammation are two molecular mechanisms of multifidus injury and atrophy after posterior lumbar surgery (Figure 2). The molecular mechanism of muscle injury is very complex [52]. Up to now, the cellular or molecular mechanism of multifidus injury is not completely clear.

The inflammatory reaction of multifidus muscle is mostly related to IL-1 *β*, tumor necrosis factor, and IL-10. According to one hypothesis, increased expression of proinflammatory cytokine and tumor necrosis factor-*α* (TNF-*α*) is associated with muscle atrophy. TNF-*α* affects myoblast differentiation and fiber degradation. TNF-*α* expression increased with intervertebral disc injury and affect axon conduction. Hodges et al. found that after intervertebral disc lesion, muscle fibrosis appeared and the TNF-*α* expression in muscle is increased [53].

James et al. [54] conducted a case-control study on mice and found that the levels of IL-1, tumor necrosis factor, IL-10, adiponectin, and leptin were lower in the sports group. The results indicated that intervertebral disc degeneration could lead to the imbalance of active inflammatory pathways in multifidus. In addition, these changes are related to the severity of intervertebral disc degeneration and can be prevented by physical exercise.

Recent animal studies [55] have found that local inflammatory dysfunction is a new mechanism to explain fat and connective tissue accumulation in multifidus muscle during disc degeneration and injury. James examined whether there were differences in the expression of inflammatory genes in multifidus between individuals with low and high intramuscular fat content to test whether there was a similar mechanism in humans. It was found that the expression of TNF in multifidus was higher in the participants with a higher degree of fat infiltration. These results support the hypothesis that intervertebral disc degeneration is associated with maladjustment of local spinous muscle inflammation.

Evidence showed that estrogen has strong antioxidant activity, which may maintain membrane stability and limit creatine kinase leakage from damaged muscles [56]. In Yang et al.'s study, estrogen protects intervertebral disc cells from apoptosis by inhibiting inflammatory cytokines IL-1*β* and TNF-*α* [57].

For the oxidative stress process, skeletal muscle pathology is mainly attributed to muscle cell membrane damage. The injury is often related to the unregulated influx of calcium through membrane lesions, which includes (1) activation of proteases and hydrolases that cause muscle injury, (2) activation of enzymes that drive muscle and immune cells to participate in injury and repair of mitogen and motilin, and (3) protein-protein interactions that promote membrane repair. At present, there is no specific cellular or molecular mechanism of multifidus injury [58]. In addition, the accumulation of free radicals caused by damage stimulation can also activate the proteolytic systems, thus, resulting in the increased protein degradation and reduced protein synthesis, which eventually lead to muscle atrophy [59].

Histochemical and pathological analysis of patients with idiopathic scoliosis showed necrosis, fibrosis, and fatty degeneration of paravertebral muscle. Compared with the control group, severe muscle injury and oxidative stress were increased in patients, and abnormal myogenesis was observed. The increased oxidative stress reaction can lead to muscle apoptosis and dysmyogenesis, which may be related to the pathological changes of idiopathic scoliosis and participate in the development and idiopathic scoliosis [60].

Dzik et al. [61] studied the changes in antioxidant enzyme activity and vitamin D receptor in paravertebral muscles with different serum vitamin D concentrations. Superoxide dismutase and glutathione peroxidase activities in the vitamin D deficiency group were significantly higher than those in the supplemented group. In vitamin D supplemented participants, lipid and protein-free radical damage markers were weakened, and the patients with high serum vitamin D concentration had stronger antioxidant capacity.

In another study [62], researchers found that vitamin D deficiency causes muscle atrophy. Vitamin D deficiency is associated with increased oxidative stress, muscle atrophy, and decreased mitochondrial function in multifidus muscle, leading to worse recovery after surgery in patients with vitamin D deficiency.

Ascorbic acid may protect multifidus muscle after operation. Tang et al. assessed the inflammation, steatosis, and fibrosis of muscle by quantitative real-time polymerase chain reaction, histological, and immunohistochemical analysis. It was found that the marker genes and scores of fibrosis and steatosis in the ascorbic acid group were significantly lower than those in the control group at 14 and 28 days after operation. It was suggested that ascorbic acid could reduce the oxidative stress and inflammatory reaction of multifidus muscle after operation [63].

Severe muscle degeneration, inflammation, and decreased blood vessels are commonly observed in biopsies of people with lumbar lesions compared to normative data. Active muscle degeneration suggests that muscle tissue change is more complex than simple atrophy [64].

## 5. Clinical Index of Multifidus Injury (Figure 3)

### 5.1. Inflammatory Index

Linzer et al. [11] chose myoglobin and creatine kinase levels as muscle injury indicators and IL-6 and C-reactive protein as systemic inflammatory indicators. In muscle injury-related research, all indexes were detected before and 1, 3, and 7 days after operation.

### 5.2. MRI

After lumbar spine surgery, MRI showed that most of the patients had fat infiltration in different degrees, and the cross-sectional area of paravertebral muscle was also decreased compared with that before operation. In Wu et al.'s study, the mean T2 signal intensity ratio of MRI increased one year after surgery [65]. The ratio of fat infiltration rate of cross-sectional area is a common index of multifidus injury. Also, Urrutia et al. [66] pointed out in the imaging study that single segment fat infiltration detection and cross-section cannot represent the degeneration of the whole lumbar spine, and multisegmental paravertebral muscle evaluation should be adopted in the study of spinal degeneration. As for multifidus fatty evaluation, Li et al. developed a measurement system for automatic segmentation of multifidus and erector spinae in MRI images based on the deep neural network [67], and Shahidi et al. used custom-written MATLAB software and two-term Gaussian model to calculate fat signal fraction [4], which avoid human factors on the calculation of fat infiltration rate.

### 5.3. Intraoperative Biopsy [68]

For intraoperative biopsy [69], the atrophic muscles were observed on preoperative imaging. During the operation, tissue scissors and other instruments are used to take atrophic muscles out, and the biopsy should be sent to the laboratory for the frozen section of atomized liquid immediately. HE staining [70] can be used to observe the regularity of muscle fiber arrangement, the clarity of muscle transverse striation, and granulation tissue infiltration. After silver staining [71], the number of branches of nerve endings and the number of muscle fibers innervated by nerves could be observed. The distribution and type of muscle fibers can be observed by ATPase staining [72], and the content and distribution of lipid in tissues can be estimated by red oil O staining [73]. Wajchenberg et al. [74] detected the fat content of the samples, which can quantitatively compare the differences between the samples.

However, there is no accepted histopathological reference value in paraspinal muscle biopsy. Zimmermann et al. used histological staining and respiratory chain enzyme biochemical analysis methods to analyze biopsy of multifidus muscle in 20 healthy subjects. It was found that the staining showed incomplete myopathy characteristics, such as increased fiber size variability, type 1 hypertrophy, intramuscular fibrosis, and fat tissue replacement. The positive rate of acid phosphatase reaction was 35%, and the changes in mitochondria were obvious. According to Zimmermann et al., it is easy to be misunderstood as myopathy due to the increased variability of morphological details. The incomplete myopathy characteristics of complex I, cytochrome c oxidase, and citrate synthase and the decrease of oxidase activity should consider as normal changes when analyzing paravertebral muscles [75].

### 5.4. Other

In addition, shear wave elastography can evaluate muscle stiffness noninvasively [76]. Ultrasonic muscle quantification can detect the degree of muscle atrophy [77]. Airaksinen et al. evaluated lumbar muscle density by computed tomography [78].

## 6. Recovery of Multifidus Function

Multifidus is an important factor to maintain spinal stability. The rest after paravertebral muscle fatigue can make the activity of paravertebral muscle decrease gradually [79]. The damaged multifidus can be repaired and activated in various ways. Clinicians should guide the choice of patients' recovery mode through professional knowledge [80]. Freeman et al. [81] found that the degree of multifidus atrophy and fat infiltration decreased in patients with one-year continuous multifidus exercise. Kliziene et al. [82] found that after eight months of core stability training, such as sit-ups, spinal bridging, and leg kick, the cross-sectional area of multifidus muscle increased by 22%. In the prospective randomized controlled study of Mannion et al. [15, 83], the isometric muscle strength, endurance, and fatigue resistance of the lumbar and dorsal muscles were significantly enhanced in patients undergoing rehabilitation exercise than those before treatment. Some experts believe that the increase of length and cross-sectional area of muscle fibers can make more nerve innervation needed after muscle training. Subsequently, new endplates will appear in large numbers to reinnervate the damaged muscles.

Besides, back stretching can improve the blood flow of lumbar muscles and effectively relieve the lower back pain related to muscle ischemia [84]. Kumamoto et al. studied the changes of oxyhemoglobin and electromyography in standing stretching exercise and found that standing stretching can improve hemodynamic performance, but excessive exercise will lead to a decrease of hemodynamic changes. Though patients with muscle ischemia should be more cautious when doing such exercises [85]. Besides that, based on taking protective measures, older adults can also benefit from exercise [86], and the inflammatory indexes of low back pain patients will decrease after exercise [87].

## 7. Summary

Multifidus is an indispensable link to maintain spinal stability. The long-term damage of chronic lumbar degenerative diseases and the direct damage of lumbar surgery are the main causes of multifidus injury. Oxidative stress and inflammation are two molecular mechanisms of multifidus injury and atrophy after posterior lumbar surgery. Minimally invasive spinal surgery is effective in protecting the postoperative function of multifidus muscle. To avoid further atrophy of multifidus and progressive degeneration of the lumbar spine, patients after lumbar surgery and patients with chronic low back pain should actively carry out long-term rehabilitation exercise of the lumbar muscles. At present, there are still some problems that need to be further studied. Such as how to avoid the disuse atrophy of paravertebral muscles as far as possible in the use of external fixation such as waist supporter after lumbar surgery, and the imaging quantitative evaluation criteria of multifidus atrophy need further research and discussion. Monitoring oxidative stress and vitamin D receptor protein content may help to further study the mechanism of vitamin D in the muscle recovery process.

## Figures and Tables

**Figure 3:**
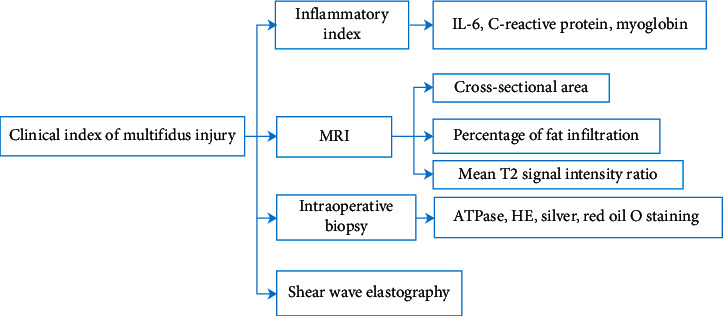
Clinical index of multifidus injury. IL: interleukin; HE: hematoxylin-eosin staining.
